# Development and preliminary psychometric evaluation of a spectacle trial-wear questionnaire for distance single-vision prescriptions

**DOI:** 10.3389/fmed.2026.1935095

**Published:** 2026-10-05

**Authors:** Meina Huang, Zheyi Chen

**Affiliations:** National Clinical Research Center for Ocular Diseases, Eye Hospital, Wenzhou Medical University, Wenzhou, China

**Keywords:** content validity, distance single-vision spectacles, patient-reported outcome measure, psychometrics, refractive care, spectacle non-tolerance, trial wear

## Abstract

**Background:**

Existing vision questionnaires were not designed to standardize immediate patient appraisal of a distance single-vision spectacle prescription during trial wear. This study developed and preliminarily evaluated the Adult Spectacle Trial-Wearing Experience Scale (ASTWES) for this decision point.

**Methods:**

Candidate items were informed by a structured literature search and interviews with eye-care and dispensing professionals, refined through two Delphi rounds, cognitively debriefed with 10 target patients, and piloted in 150 adults. Item reduction used corrected item-total correlations and principal-axis factoring with promax rotation. The independent formal psychometric evaluation sample comprised 393 adults within the prespecified 18–35-year age range; ordinal confirmatory factor analysis (CFA), robust maximum-likelihood sensitivity analysis, and reliability coefficients were used. An eligible subsample of 98 participants provided a 1-month satisfaction rating for exploratory association analyses.

**Results:**

The final questionnaire contained 9 items in 3 correlated domains: clarity and focus, comfort and stability, and environmental adaptation. Retained pilot-item loadings ranged from 0.694 to 0.890. The correlated three-factor model was supported under both CFA estimators, whereas a single-factor model fit poorly. Subscale Cronbach's alpha values ranged from 0.769 to 0.803. Omega hierarchical was 0.641, favoring subscale-first interpretation. The overall mean item score correlated with 1-month satisfaction (Spearman rho = 0.753, *p* < 0.001); its exploratory AUC was 0.850 (bootstrap 95% CI 0.772–0.918).

**Conclusions:**

ASTWES is a concise candidate questionnaire with preliminary evidence of a correlated three-domain structure, acceptable internal consistency, and short-term clinical association in Chinese adults aged 18–35 years. Further independent patient-centered content evaluation and external validation are needed before routine use or fixed decision thresholds are considered.

## Introduction

Refractive error remains a major cause of visual disability, and spectacles are a widely used and cost-effective correction modality (1–3). The burden remains substantial in early adulthood: in the Raine cohort, myopia prevalence increased from 25.8% at age 20 years to 33.2% at age 28 years (4). Because refractive care is judged not only by acuity but also by whether the prescribed correction is acceptable to the patient, subjective appraisal is relevant to care quality and value (5, 6).

The narrower clinical problem is spectacle non-tolerance and prescription acceptance. Studies of non-tolerance after dispensing describe blur, difficulty refocusing, dizziness, instability, altered spatial judgment, and discomfort after a new prescription or lens change (7–11). These reports mainly concern complaints recognized after dispensing, whereas experimental work shows that spatial distortion, image minification, and acceptance of small refractive changes can also be experienced during initial exposure (12–14). Perceptual adaptation to a new astigmatic correction may then continue beyond the initial encounter (15). The present study therefore focuses specifically on the immediate trial-wear window, not on later habitual-wear adaptation. At this decision point, symptoms are commonly elicited through unstructured questioning rather than a context-specific patient-reported instrument, limiting standardized documentation and comparison across patients or visits.

The construct of interest in the present study is immediate spectacle trial-wearing appraisal: the patient's self-reported appraisal, after short trial wear with the final candidate prescription, of (1) visual clarity across relevant viewing distances, (2) comfort and visual stability during ordinary head and eye movements, and (3) adaptation to routine environmental and ambulatory tasks encountered during trial wear. This construct is related to, but distinct from, broader vision-related quality of life, long-term spectacle satisfaction, and patient experience of service delivery. In other words, ASTWES is intended as a patient-reported outcome measure of immediate subjective optical appraisal during dispensing, not as a patient-reported experience measure of clinic service.

Existing instruments do not fully address this context of use. NEI-VFQ-25 and VF-14 primarily assess longer-term functional vision and day-to-day activity limitations (16, 17). QIRC compares the quality-of-life impact of different refractive correction modalities rather than the immediate appraisal of a candidate prescription (18). QoV captures optical symptoms such as glare, haloes, blur, and diplopia, but was not designed for the short trial-wearing window in outpatient dispensing (19). Our structured item-generation search did not identify a brief questionnaire designed specifically for immediate trial wear of conventional distance single-vision spectacles.

The present work was designed as an initial development and psychometric evaluation study informed by established recommendations for patient-reported questionnaire development and measurement-property reporting (20–27). It examined construct definition, professional and expert review of candidate content, pilot patient feedback, structural validity, internal consistency, exploratory subgroup stability, and short-term clinical association. The intended contribution is a candidate instrument and scoring framework for a specific dispensing decision point, rather than a completed validation program.

The conceptual framework for ASTWES was grounded in recurring symptom domains in spectacle non-tolerance and adaptation literature (7–10), the dispensing context described by Donabedian's process perspective (5), and professional interviews focused on what patients commonly report during short in-clinic trial wear. The resulting candidate domains were clarity and focus, comfort and stability, and environmental adaptation. The study aimed to develop a brief questionnaire around these domains and determine whether its structure was psychometrically plausible in pilot and formal samples of Chinese adults aged 18–35 years.

## Methods

### Study design and scope

This was a single-center, multi-stage scale development study conducted at the Eye Hospital of Wenzhou Medical University. Pilot recruitment occurred from April to June 2025; after pilot item reduction and finalization of V3, a separate formal sample was recruited from July to October 2025. The pilot and formal samples were independent, and no pilot participant entered formal testing. Both samples were recruited from the outpatient optometry clinic using non-probability quota sampling: eligible patients were enrolled until prespecified targets for sex and broad refractive category were filled. The research comprised: (1) construct clarification and literature-informed item generation; (2) professional interviews; (3) patient cognitive debriefing with open-ended concept elicitation; (4) Delphi consultation; (5) pilot testing and item reduction; (6) formal psychometric testing; and (7) exploratory 1-month follow-up. Participant flow and the relationship among analytic samples are shown in Figure 1. Ethical approval was granted by the Research Ethics Committee of the Affiliated Eye Hospital of Wenzhou Medical University (approval number: 2024-225-K-186-01). All participants provided written informed consent, and the study adhered to the Declaration of Helsinki.

**Figure 1:**
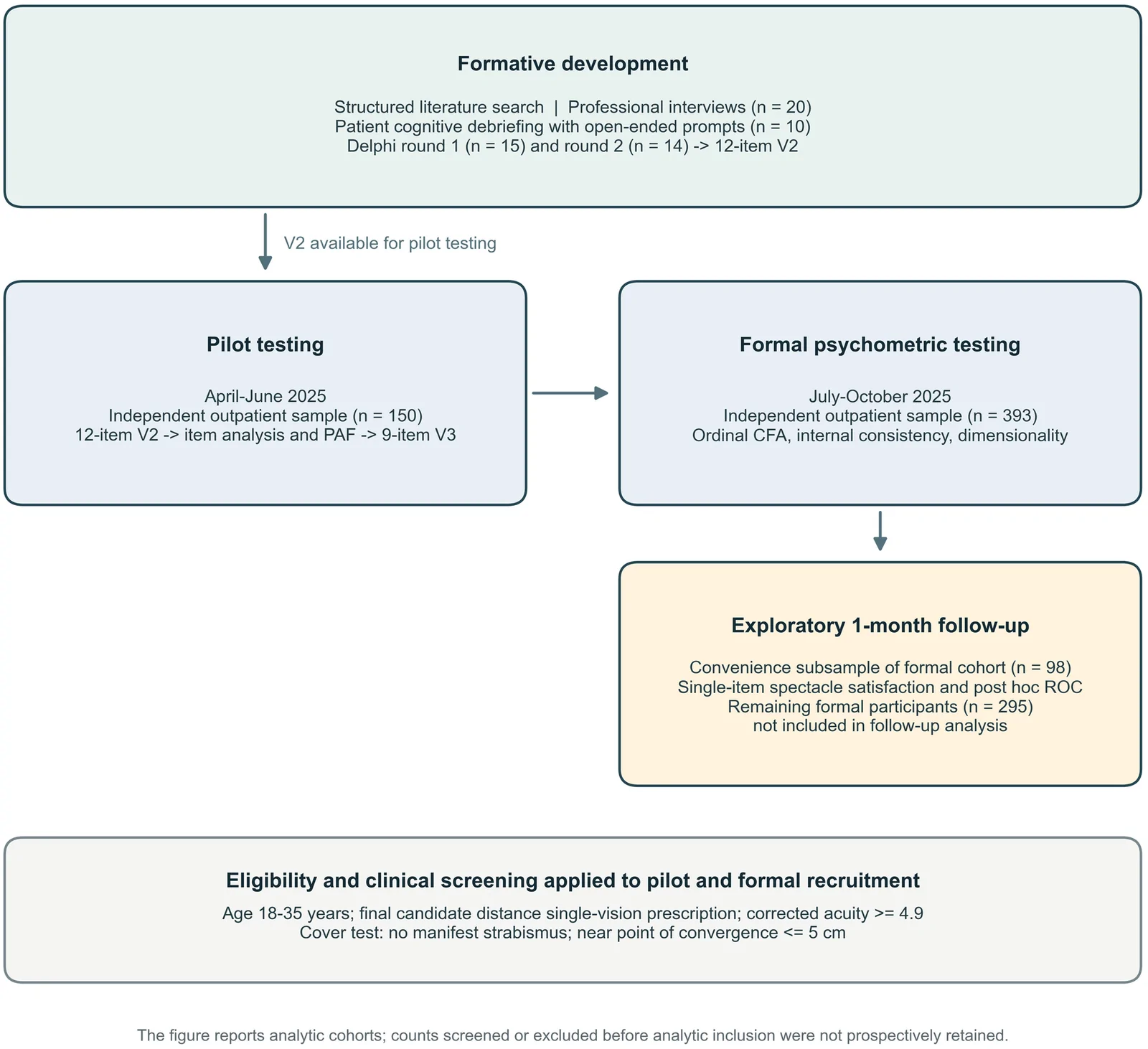
Participant flow across questionnaire development and psychometric evaluation. The pilot and formal samples were independently recruited, and no pilot participant entered formal testing. The 1-month follow-up was a convenience subsample of the formal cohort. Counts screened or excluded before creation of the analytic cohorts were not prospectively retained and therefore are not shown.

The present manuscript reports evidence on construct definition, patient, professional, and expert review of candidate content, structural validity, internal consistency, exploratory dimensionality, exploratory subgroup stability across sex and age bands, and association with a pragmatic 1-month satisfaction anchor.

### Construct definition and context of use

ASTWES was designed for adults trial-wearing a final candidate prescription for conventional distance single-vision spectacles in an outpatient dispensing workflow. Questionnaire administration followed a trial that typically lasted approximately 20 min. Individual start and end times were not recorded continuously, so a sample mean and standard deviation for trial duration were unavailable. An optometry intern assigned to the study observed each participant throughout trial wear. The route began outside the examination room, continued along a relatively long clinic corridor, included the adjacent stair section connecting the upper and lower floors, and extended into a hall containing visual targets at near and far distances and areas of differing ambient brightness. Participants viewed targets at different distances, walked on level ground, and ascended and descended steps before returning to the clinic; the intern confirmed completion on return. The physical route and required task types were consistent, but an exact walking distance, stair count, or number of repetitions was not prescribed. Higher scores reflect better immediate appraisal of the candidate prescription. Subscale scores were treated as the primary scoring units; an overall mean item score was explored secondarily because the final domains were conceptually related and clinically co-occurred during trial wear.

Although the candidate prescription was for distance single-vision spectacles, near-vision and refocusing items were included because non-presbyopic adults ordinarily continue to wear the distance prescription while viewing near targets and changing fixation distance. The environmental-adaptation items refer to immediate optical and spatial responses across the viewing-distance, ambient-brightness, level-walking, and stair-negotiation conditions encountered on the clinic route, rather than long-term neural adaptation to a new prescription.

### Item pool generation

#### Structured literature search

A structured literature search was undertaken to map existing instruments and identify unmet measurement needs, rather than as a standalone systematic or scoping review. PubMed, Web of Science, Embase, CNKI, Wanfang, and VIP were searched for literature published from 1 January 2000 to 31 December 2024. Search terms combined refractive-care context (“optometry”, “spectacles”, “dispensing”, “trial wear”, “visual quality”, “experience”, and “scale”) and their Chinese equivalents. Candidate sources included vision questionnaires, spectacle non-tolerance studies, dispensing studies, and clinically relevant reports describing symptoms or tasks encountered during trial wear. Two investigators screened potentially relevant records and extracted recurring symptom domains, task contexts, wording patterns, and available measurement-property evidence. Because full PRISMA-level screening records were not retained, the search is reported as an item-generation literature search; its databases, terms, and eligibility rules are provided in Supplementary Table S1.

#### Semi-structured professional interviews

Purposive semi-structured interviews were conducted with optometrists/ophthalmic dispensers (*n* = 10) and dispensing consultants (*n* = 10) who routinely observed short trial wear in clinic. All 20 interviewees were staff members of the study department at the study center and were selected because their direct duties involved trial wear or spectacle dispensing. Critical Incident Technique prompts were used to elicit recurrent patient complaints, behaviors, and explanations observed during trial wear (28). The research team summarized the interview material descriptively into recurrent complaint, behavior, and task categories (29). This was a formative item-generation exercise rather than a standalone qualitative study; no formal codebook-based thematic analysis or saturation analysis was undertaken. These professional interviews informed the context of use, candidate wording, and initial domain framework.

#### Patient cognitive debriefing and concept elicitation

Immediately before pilot testing, 10 adults who met the intended outpatient population and prescription-context criteria were recruited from the optometry clinic for brief individual cognitive interviews on the V2 questionnaire after trial wear of a final candidate distance single-vision prescription. Each interview lasted no more than approximately 15 min. A combined think-aloud and verbal-probing approach was used to examine the relevance and interpretation of each item, the five response options, the immediate trial-wear reference period, and whether any important experience was absent from the candidate framework. Open-ended prompts asked which visual changes or difficulties mattered when deciding whether the prescription was acceptable. Particular attention was paid to the answerability of comfort, environmental adaptation, level-walking, and stair-negotiation items within the short trial-wear procedure. Contemporaneous field notes were taken and summarized after each interview. The review supported the use of concrete viewing examples, the immediate trial-wear reference period, and direct confirmation that walking and stair tasks had been completed. A separate demographic and refractive-characteristics table was not retained for this 10-person formative group and was not reconstructed retrospectively.

#### Research-team synthesis

The research team integrated evidence from the literature search, professional interviews, patient debriefing, and expert review to define the construct, context of use, response format, and provisional framework. The initial pool (V1) contained 24 items across four provisional domains. Because the instrument was intended for a brief outpatient workflow, emphasis was placed on relevance to short in-clinic trial wear, task feasibility, and concise wording. Patient feedback was incorporated into the research team's review of item wording, administration, and short-trial answerability.

### Delphi expert consultation

A two-round Delphi consultation was performed with 15 experts purposively selected from six institutions for relevant clinical or methodological expertise (30). The panel comprised optometry/refraction (*n* = 6), ophthalmology (*n* = 4), ophthalmic dispensing (*n* = 3), and statistics/scale methodology (*n* = 2). Nine experts held senior and six held intermediate professional titles; their mean professional experience was 15.3 ± 3.7 years (range 9–22). Experts rated each item for importance, relevance, and operability and provided qualitative comments. Round 2 included feedback on round-1 results and revision notes. De-identified panel characteristics are reported in Supplementary Table S6.

Protocol-defined indicators were response rate >80%, authority coefficient (Cr) >0.70, coefficient of variation <0.25, and item-level content validity index (I-CVI) >0.78 (31, 32). Kendall's W was used to summarize round-wise agreement. Item-level I-CVI, importance means and coefficients of variation were recalculated from the two rounds of expert ratings. Decisions were checked against the V1-to-V2 item audit trail and considered relevance, importance, dispersion, redundancy, short-trial answerability, and domain coverage jointly.

### Pilot testing and item reduction

Pilot participants were adults aged 18–35 years attending the outpatient optometry clinic and requiring spectacle dispensing. Inclusion criteria were age 18–35 years, completion of clinical refraction, spectacle-corrected visual acuity ≥4.9 in Chinese standard logarithmic notation (decimal acuity ≥0.8; approximately Snellen 20/25 or logMAR 0.10), and ability to complete the questionnaire independently. The upper age limit was selected as a pragmatic design restriction to reduce the influence of age-related accommodative decline and emerging near-add requirements on responses to the near-vision and refocusing items. Routine clinical screening included cover testing and near point of convergence assessment. Participants were excluded if manifest strabismus was detected on cover testing or if the measured near point of convergence exceeded 5 cm. To reduce major binocular confounding, participants with anisometropia >1.00 D spherical-equivalent inter-eye difference were excluded, along with those with severe ocular disease, severe systemic disease, or inability to complete the questionnaire.

Questionnaires were administered only after the final prescription to be dispensed had been confirmed. If a prescription was adjusted during trial wear, the earlier trial was not scored. Participants completed the questionnaire after approximately 20 min of supervised trial wear. The observing intern monitored completion of the clinic route described above, ensuring that every participant viewed near and distant targets, experienced the available brightness variation, walked on level ground, and negotiated steps; completion was confirmed when the participant returned. No exact walking distance, stair count, or repetition count was required. Investigators provided standardized instructions but did not guide item responses. Quantitative item performance and the research team's content review were jointly used for V2-to-V3 item reduction.

During questionnaire drafting, two clarity items exchanged labels before field administration. To avoid ambiguity, all pilot analyses in this manuscript use the administered V2 numbering: in the pilot questionnaire A3 referred to intermediate-distance clarity and A4 referred to rapid refocusing. After item reduction, the retained rapid-refocusing item was renumbered A3 in the final V3 scale.

Item reduction used corrected item-total correlation (CITC), change in Cronbach's alpha after item deletion, and common factor analysis (33, 34). Principal-axis factoring with oblique rotation (promax) was used because correlated latent dimensions were expected *a priori*. Factor retention was informed by the conceptual framework, scree inspection, and parallel analysis. For the retained V3 items, loadings ≥0.30 with a theoretically coherent three-factor pattern were considered adequate for an initial development stage (23, 24).

The pilot target of 150 participants exceeded five participants per item for the 24-item initial pool. The formal target was at least 10 participants per retained item and sufficient observations for the prespecified three-factor CFA; the eligible formal analytic sample contained 393 participants.

### Scoring and missing responses

Each item is scored from 1 (strongly disagree) to 5 (strongly agree). Each three-item subscale score is the arithmetic mean of its three constituent items and therefore retains the 1–5 metric. The secondary overall score is the mean of all nine items, also on the 1–5 metric. A subscale score is not calculated if any of its three responses is missing, and the overall score is not calculated unless all nine responses are present. No item-level imputation is recommended. The study datasets contained no missing item responses.

### Formal psychometric testing

The formal psychometric sample comprised 393 eligible adults recruited independently from the same clinic after completion of the pilot phase. The same non-probability quota approach was used to balance sex and broad refractive case-mix. Here, case-mix denotes the routine clinician-assigned recruitment labels describing the principal refractive presentation (low, moderate, or high myopia; astigmatism-dominant; hyperopia; or emmetropia/mild refractive error) and first-time/initial vs. previous/replacement spectacle use. These labels were used for recruitment balance and descriptive reporting, not as randomized sampling strata or adjudicated research diagnoses. No retrospective dioptric cutoffs or study-eye selection rule were imposed on the labels; actual signed spherical and cylindrical powers were therefore reported separately. Sex and age were recorded directly, and the prescription powers were parsed from the information embedded in the labels for descriptive reporting.

Internal consistency was assessed with Cronbach's alpha and Spearman-Brown split-half reliability (35). The overall scale used an odd-even item split; for each three-item subscale, the middle item was correlated with the sum of the first and third items before Spearman-Brown correction. Subscale scores were prespecified as the primary interpretation units. Overall-score dimensionality was examined using McDonald's omega, omega hierarchical, explained common variance (ECV), and percentage of uncontaminated correlations (PUC) (36). Because item responses were ordinal, the primary correlated three-factor and single-factor CFA models used weighted least squares mean- and variance-adjusted estimation (WLSMV) in lavaan. Robust maximum likelihood (MLR) was used as a sensitivity analysis to determine whether conclusions depended on estimator choice. Model evaluation emphasized CFI/TLI >0.95, RMSEA <0.06, and SRMR <0.08, considered jointly rather than as rigid pass/fail rules (23, 24, 37).

Exploratory multi-group CFA examined configural, metric, and scalar models across sex and age bands (18–25 vs. 26–35 years). Ordinal WLSMV subgroup models were attempted but were not estimable because several item-by-group response categories were empty. Robust MLR treating the five response categories as approximately continuous was therefore used only as a sensitivity analysis of subgroup stability. Changes of CFI ≤0.01, RMSEA ≤.015, and SRMR ≤0.03 for metric and ≤0.01 for scalar models were considered supportive (38). Although refractive powers and first-time wear could be derived from routine case-mix labels for descriptive reporting, these variables were not used for invariance modelling because they were not stored as prespecified structured grouping variables.

### Exploratory 1-month follow-up

The 1-month follow-up sample was a convenience subsample of 98 eligible participants from the formal cohort who had completed baseline ASTWES, obtained the prescribed spectacles, could be contacted, and completed the approximately 1-month assessment by telephone or review visit. Participants rated their overall satisfaction with the dispensed spectacles on a single 5-point ordinal item, from 1 (lowest satisfaction) to 5 (highest satisfaction). This rating was used as a pragmatic clinical anchor for short-term association rather than as a standalone validation instrument or a criterion standard. Analyses included Spearman correlations between baseline ASTWES scores and follow-up satisfaction and a *post hoc* ROC description for satisfied (scores 4–5) vs. not satisfied (scores 1–3) (39, 40). A stratified percentile bootstrap with 5,000 resamples estimated the 95% confidence interval for AUC. The sample-derived Youden value was treated as descriptive and was not proposed as a clinical threshold (41–45).

### Statistical analysis

Data were double-entered and logic-checked. Descriptive statistics, item analysis, factor analysis, reliability analysis, CFA, subgroup sensitivity analysis, correlation analysis, and ROC analysis were conducted in SPSS and R, including psych and lavaan. There were no missing item responses in the pilot, formal, or follow-up analytic files; no imputation was performed. Continuous variables are presented as mean ± standard deviation unless otherwise stated. Two-sided *p* < 0.05 was considered statistically significant.

## Results

### Sample characteristics

Table 1 summarises the three analytic samples: 150 participants recruited for pilot testing from April to June 2025, a separate sample of 393 recruited for formal testing from July to October 2025, and a convenience follow-up subsample of 98 from the formal cohort. Pilot and formal samples were independent, balanced by sex, and centered in the mid-20s. Routine case-mix labels indicated that moderate and low myopia predominated. Mean spherical power was −3.66 ± 2.49 D in the pilot sample, −2.67 ± 2.52 D in the formal sample, and −2.57 ± 2.37 D in the follow-up sample; corresponding mean cylindrical powers were −0.29 ± 0.50 D, −0.49 ± 0.63 D, and −0.41 ± 0.60 D.

**Table 1:** Sample characteristics across study phases.

Characteristic	Pilot testing	Formal testing	Follow-up subsample
N	150	393	98
Recruitment period	April-June 2025	July-October 2025	1 month after formal baseline
Age, years	25.9 ± 3.6	26.0 ± 3.7	25.6 ± 3.7
Observed age range, years	19–33	18–35	18–35
Sex, male/female	75/75	198/195	45/53
First-time or initial spectacle wear, *n* (%)	58 (38.7)	87 (22.1)	23 (23.5)
Low myopia, *n* (%)	31 (20.7)	136 (34.6)	29 (29.6)
Moderate myopia, *n* (%)	47 (31.3)	171 (43.5)	49 (50.0)
High myopia, *n* (%)	34 (22.7)	37 (9.4)	7 (7.1)
Astigmatism-dominant label, *n* (%)	26 (17.3)	21 (5.3)	4 (4.1)
Hyperopia, *n* (%)	1 (0.7)	18 (4.6)	5 (5.1)
Emmetropia/mild refractive error, *n* (%)	0 (0.0)	10 (2.5)	4 (4.1)
Other case-mix labels, *n* (%)	11 (7.3)	0 (0.0)	0 (0.0)
Spherical power, D	−3.66 ± 2.49	−2.67 ± 2.52	−2.57 ± 2.37
Cylindrical power, D	−0.29 ± 0.50	−0.49 ± 0.63	−0.41 ± 0.60

For all three samples, first-time wear and refractive case-mix categories were derived from routine recruitment labels. Signed spherical and cylindrical powers were parsed from the prescription information embedded in the patient-type labels; an unreported component was coded as 0 D, and the final value was used when a change from an earlier to a later power was recorded.

### Patient input during item development

Ten target patients participated in brief pre-pilot cognitive debriefing with open-ended concept prompts. They reviewed item relevance and interpretation, response options, the immediate reference period, and the answerability of tasks during short trial wear. The field-note summaries covered distance and near clarity, refocusing, binocular comfort, image stability, and ambulatory adaptation. Concrete examples for distance and near viewing were retained, and the supervised route directly incorporated different viewing distances, ambient-brightness variation, level walking, and stair negotiation. The patient-review process is summarized in Supplementary Table S4.

### Delphi consultation and item reduction

Fifteen experts from six institutions participated in round 1 and 14 in round 2. Professional backgrounds included optometry/refraction (*n* = 6), ophthalmology (*n* = 4), ophthalmic dispensing (*n* = 3), and statistics/scale methodology (*n* = 2). Nine held senior and six held intermediate professional titles, with 15.3 ± 3.7 years of professional experience (range 9–22). The mean authority coefficient was 0.88 (Ca = 0.90, Cs = 0.87). Kendall's W increased from 0.17 in round 1 to 0.36 in round 2, indicating greater, although still modest, agreement in item-importance rankings. Delphi summary indicators are presented in Table 2. The I-CVI ranges were 0.80–1.00 in round 1 and 0.714–1.00 in round 2; S-CVI/Ave values were 0.94 and 0.95. The only round-2 item below 0.78 was V1–24 (turning while maintaining clear, continuous vision; I-CVI = 0.714), which was deleted because of limited short-trial answerability and overlap with more representative movement-stability items. All V1 source items retained or merged into V2 met the round-2 I-CVI criterion, and their round-2 importance coefficients of variation were below 0.25. A third Delphi round was not conducted because the retained content met the prespecified item-level relevance and dispersion criteria; remaining disagreement concerned items already designated for deletion because of redundancy or task feasibility. Item-level results and the complete V1-to-V2 decision trail are provided in Supplementary Table S5; de-identified expert characteristics are provided in Supplementary Table S6.

**Table 2:** Delphi summary indicators.

Indicator	Value	Comment
Number of experts	15	Multidisciplinary
Rounds completed	2	15 responses in round 1; 14 in round 2
Round-1 response rate	100.0%	15/15
Round-2 response rate	93.3%	14/15
Mean authority coefficient (Cr)	0.88	Ca = 0.90; Cs = 0.87
Round-1 Kendall's W	0.17	chi-square = 57.8, *p* < 0.001
Round-2 Kendall's W	0.36	chi-square = 117.4, *p* < 0.001
Round-1 S-CVI/Ave	0.94	I-CVI range 0.80–1.00
Round-2 S-CVI/Ave	0.95	I-CVI range 0.71–1.00
V1 to V2 reduction	24 to 12 items	9 deletions and 3 mergers/rewordings

As documented in the original development report, the V1 pool was reduced to a 12-item pilot questionnaire (V2) organized into three provisional domains: clarity and focus (A1-A4), comfort and stability (B1-B4), and environmental adaptation (C1-C4).

### Pilot testing: item analysis and common factor structure

Pilot item analysis identified 3 weak items. Using the administered V2 numbering, A3 (intermediate-distance clarity), B4 (a compound symptom item covering dizziness, headache, and neck discomfort), and C4 (adaptation to light transitions) had CITC values of 0.267, 0.245, and 0.233, respectively (Table 3), and weaker factor performance than the other items. A3 overlapped with the retained distance-, near-, and refocusing items; B4 combined heterogeneous symptoms already represented more specifically by the comfort and stability items; and C4 was not consistently answerable within the clinic trial environment. These psychometric and content considerations jointly supported deletion.

**Table 3:** Pilot item analysis for the administered V2 questionnaire (*n* = 150).

Item	Mean	SD	CITC	Alpha if deleted
A1	4.033	0.781	0.317	0.765
A2	4.053	0.775	0.337	0.762
A3*	4.093	0.838	0.267	0.770
A4	3.993	0.823	0.426	0.753
B1	4.093	0.846	0.532	0.741
B2	3.967	0.823	0.487	0.747
B3	3.927	0.836	0.514	0.743
B4*	4.160	0.705	0.245	0.771
C1	4.013	0.912	0.540	0.739
C2	3.920	0.815	0.488	0.746
C3	3.880	0.904	0.487	0.746
C4*	3.960	0.834	0.233	0.774

Asterisks indicate candidate items for deletion. Total-scale alpha for V2 was 0.771.

Sampling adequacy was acceptable for common factor analysis (KMO = 0.77; Bartlett chi-square =616.94, df = 66, *p* < 0.001), and parallel analysis supported a three-factor solution. In the 12-item model, retained items loaded strongly on the anticipated factors, whereas A3, B4, and C4 showed the weakest primary loadings (0.384, 0.290, and 0.483, respectively). After deleting these items, the retained 9-item structure was re-estimated using the final V3 labels, where the retained pilot item A4 was renumbered as final A3. The retained items loaded from 0.694 to 0.890, with small-to-moderate factor correlations (0.113–0.369), supporting a correlated three-domain structure. The retained-item loadings are shown in Table 4.

**Table 4:** Principal-axis factoring with promax rotation for retained V3 items (pilot sample, *n* = 150).

Item	Environmental adaptation	Comfort and stability	Clarity and focus
A1	–	–	0.806
A2	–	–	0.702
A3	–	–	0.694
B1	–	0.805	–
B2	–	0.797	–
B3	–	0.779	–
C1	0.808	–	–
C2	0.760	–	–
C3	0.890	–	–

Loadings <0.20 are omitted for clarity. Factor correlations were 0.369 between environmental adaptation and comfort/stability, 0.290 between comfort/stability and clarity/focus, and 0.113 between environmental adaptation and clarity/focus.

### Formal testing: structural validity, reliability, and exploratory subgroup stability

In the formal sample, subscale Cronbach's alpha values ranged from 0.769 to 0.803, and omega hierarchical was 0.641 (Table 5).

**Table 5:** Formal-sample reliability and exploratory general-factor indices (*n* = 393).

Scale/domain	Items	Cronbach's alpha	Split-half reliability	Additional index
Overall 9-item summary	9	0.824	0.866	omega total = 0.882; omega hierarchical = 0.641; ECV = 0.486; PUC = 0.750
Clarity and focus	3	0.803	0.787	Primary scoring unit
Comfort and stability	3	0.769	0.755	Primary scoring unit
Environmental adaptation	3	0.787	0.780	Primary scoring unit

Ordinal WLSMV CFA supported the correlated three-factor structure in the primary formal sample: chi-square = 14.605, df = 24, CFI = 1.000, TLI = 1.000, RMSEA < 0.001, and SRMR = 0.029. Standardized loadings ranged from 0.736 to 0.859. Because chi-square/df was below 1 and incremental fit indices reached the upper bound, model diagnostics were examined rather than interpreting the fit as proof of a perfect population model. Only 4 residual correlations exceeded an absolute value of 0.05, the largest absolute residual was 0.076, the largest modification index was 3.37, and no modification index exceeded 10 (Supplementary Table S3). The single-factor WLSMV alternative fit poorly. A robust MLR sensitivity analysis reached the same substantive conclusion for the correlated three-factor model (chi-square = 28.823, df = 24, CFI = 0.996, TLI = 0.994, RMSEA = 0.023, SRMR = 0.026). Model-fit indices for both estimators are summarized in Table 6.

**Table 6:** CFA models and estimator sensitivity analysis for the formal sample (*n* = 393).

Estimator and model	chi-square	df	CFI	TLI	RMSEA	SRMR
WLSMV correlated three-factor CFA	14.605	24	1.000	1.000	<0.001	0.029
WLSMV single-factor CFA	391.326	27	0.898	0.864	0.186	0.138
MLR correlated three-factor CFA	28.823	24	0.996	0.994	0.023	0.026
MLR single-factor CFA	403.571	27	0.682	0.576	0.188	0.114

In the exploratory MLR sensitivity analysis, changes in fit indices were small across configural, metric, and scalar models for sex and age bands. Across sex, Delta CFI values were +0.003 from configural to metric and −0.003 from metric to scalar. Across age bands, Delta CFI values were −0.001 from configural to metric and +0.003 from metric to scalar. Because MLR treated the five response categories as approximately continuous after ordinal subgroup models failed, these findings are interpreted as provisional subgroup-stability evidence rather than confirmation of ordinal scalar invariance. Full fit statistics and sparse-category diagnostics are reported in Supplementary Table S2.

### Exploratory association with 1-month satisfaction

The follow-up satisfaction distribution was 9 participants at score 1, 15 at score 2, 25 at score 3, 25 at score 4, and 24 at score 5. The follow-up associations and exploratory ROC results are summarized in Table 7. Baseline overall mean item score correlated strongly with 1-month satisfaction (Spearman rho = 0.753, *p* < 0.001). Domain-specific correlations were also significant: clarity and focus rho = 0.616, comfort and stability rho = 0.654, and environmental adaptation rho = 0.501 (all *p* < 0.001). For the *post hoc* dichotomy of satisfied (scores 4–5) vs. not satisfied (scores 1–3), the overall mean item score had an exploratory AUC of 0.850 (bootstrap 95% CI 0.772–0.918; Figure 2). The sample-derived Youden value was 4.22, with sensitivity 0.735 and specificity 0.796; it was not treated as a validated clinical threshold.

**Table 7:** Exploratory 1-month association analyses in the follow-up sample (*n* = 98).

Analysis	Statistic	Result
Satisfaction distribution, scores 1–5	n	9/15/25/25/24
Spearman correlation, overall mean item score vs. 1-month satisfaction	rho	0.753, *p* < 0.001
Spearman correlation, clarity and focus vs. 1-month satisfaction	rho	0.616, *p* < 0.001
Spearman correlation, comfort and stability vs. 1-month satisfaction	rho	0.654, *p* < 0.001
Spearman correlation, environmental adaptation vs. 1-month satisfaction	rho	0.501, *p* < 0.001
Post hoc overall-score ROC for satisfaction scores 4–5	AUC	0.850 (bootstrap 95% CI 0.772–0.918)
Sample-derived Youden reference point, overall mean item score	Mean item score	4.22; sensitivity 0.735; specificity 0.796

**Figure 2:**
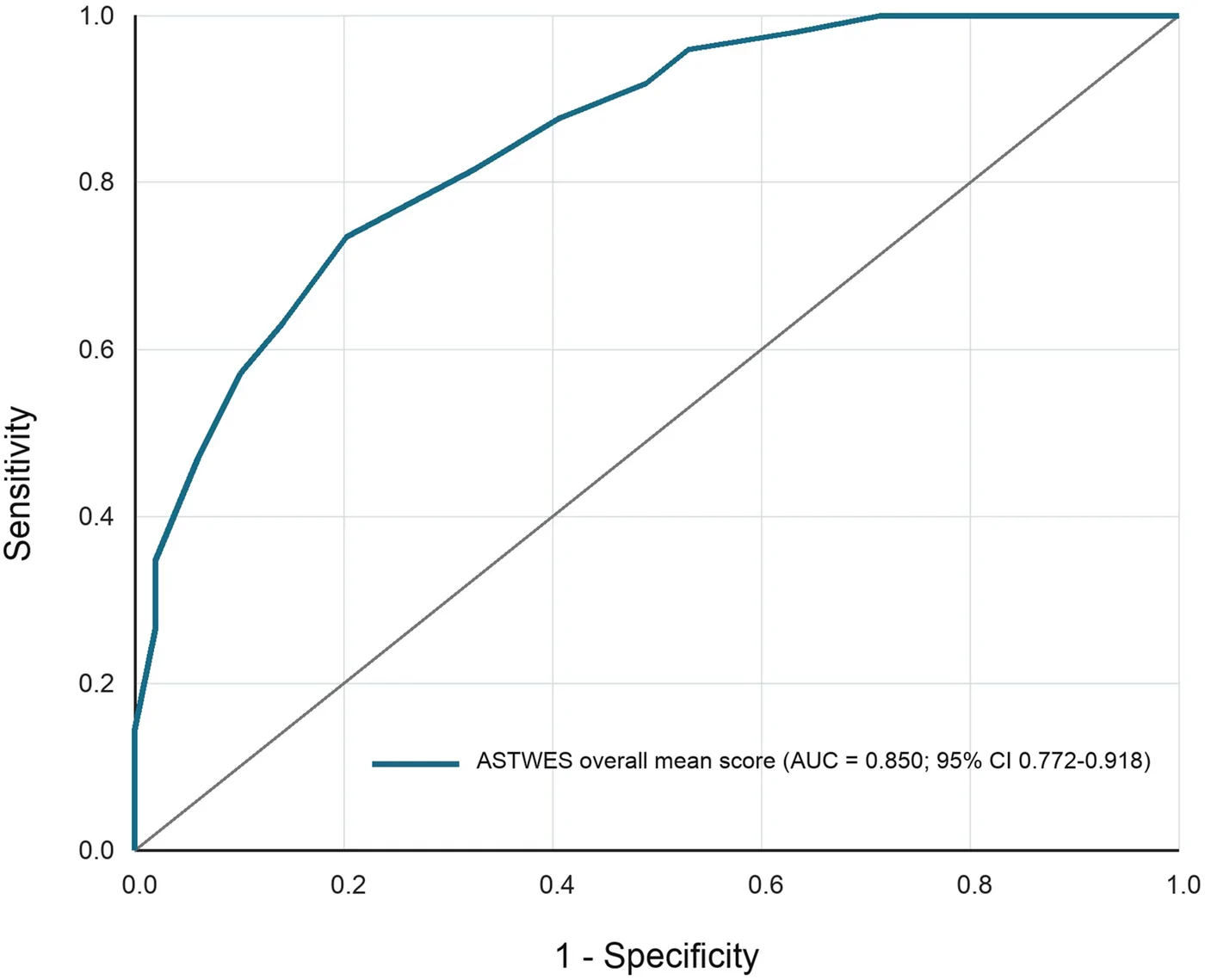
*Post hoc* ROC curve describing discrimination of 1-month satisfaction (scores 4–5) by the baseline ASTWES overall mean item score in the eligible follow-up sample (*n* = 98; AUC = 0.850; stratified-bootstrap 95% CI 0.772–0.918).

### Final scale and scoring

ASTWES V3 contains 9 items scored from 1 (strongly disagree) to 5 (strongly agree). At the current stage, the recommended interpretation is:

1. Use **subscale mean scores** as the primary summary because the final structure is multidimensional.
2. Use the **overall mean item score** only as a secondary exploratory summary.
3. Report the *post hoc* value of 4.22 descriptively only; it is not recommended for individual clinical decisions until independently validated.

Subscale and overall scores remain on the 1–5 mean-item metric. A subscale requires all three constituent responses, and the overall mean requires all nine responses; otherwise, the corresponding score is not calculated.

## Discussion

This study developed a brief questionnaire for a narrowly defined clinical context: immediate patient appraisal after trial wear of a candidate distance single-vision spectacle prescription. The findings provide preliminary evidence for a correlated three-domain structure, acceptable internal consistency, and an exploratory short-term association with 1-month satisfaction in Chinese adults aged 18–35 years.

The retained 9 items showed a coherent three-factor pattern in pilot common factor analysis. Ordinal CFA yielded near-perfect incremental fit indices, but the low residual correlations, small modification indices, and consistent MLR sensitivity analysis reduce concern that the result was created by *post hoc* residual correlations or hidden cross-loadings. Near-perfect fit may partly reflect the parsimonious 9-item structure and the relatively homogeneous sample of adults aged 18–35 years; replication in a new clinical cohort would be useful.

The item pool combined literature, professional observation, patient cognitive debriefing with open-ended concept prompts, and expert review. Patient input provided a direct check of item relevance, comprehensibility, response-option use, the immediate reference period, and short-trial answerability in the target population (21, 23–27).

Principal-axis factoring with oblique rotation produced a three-domain solution consistent with the correlated CFA structure. This method is appropriate when the aim is to estimate related latent dimensions rather than to reduce observed variables into orthogonal components (23, 24).

The scale should not be interpreted as essentially unidimensional. The single-factor model fit poorly, omega hierarchical was 0.641, and ECV was 0.486. These results favor the three subscales as the primary interpretation units (36). The overall mean item score is best used as a brief secondary summary when a global trial-wear impression is clinically useful.

The inclusion of near-vision and refocusing items does not redefine ASTWES as a near-vision or progressive-lens questionnaire. In non-presbyopic adults, a distance single-vision prescription is commonly worn while viewing a smartphone or changing fixation distance; over- or under-correction may therefore be experienced through near clarity or refocusing comfort during the same trial. Likewise, the environmental-adaptation domain captures immediate optical and spatial responses across varied viewing distances, ambient brightness, level walking, and stair negotiation, not long-term adaptation to habitual wear.

The 1-month follow-up analysis should be read as exploratory evidence of short-term clinical association rather than as a prediction model. The correlation and AUC suggest that immediate trial-wearing appraisal is related to later spectacle satisfaction in this convenience sample, but the single-item outcome, *post hoc* dichotomy, and absence of external validation preclude clinical use of a fixed threshold (41–45).

Although the name ASTWES uses the word “experience”, the instrument does not measure service experience in the PREM sense. Its items ask about immediate patient-reported optical outcomes during trial wear: clarity, comfort/stability, and environmental adaptation. ASTWES is therefore more accurately classified as a brief PROM of immediate trial-wearing appraisal than as a PREM of clinic processes. If later validated, its clinical role would be to supplement rather than replace refractive examination and professional judgment (46).

The Mandarin version is the source instrument. Table 8 presents the complete English reporting translation, and Supplementary File S1 provides the full English questionnaire followed by the Mandarin source form. The English wording is supplied for international reader comprehension; future English-language use should include forward and backward translation, reconciliation, cognitive debriefing, and cross-cultural validation (23, 24).

**Table 8:** Final ASTWES item content (English reporting translation).

Domain	Item	Reporting translation in English
Clarity and focus	A1	Viewing distant objects (e.g., signage at the end of a corridor) appears clear without distortion.
Clarity and focus	A2	Viewing near objects (e.g., a smartphone screen) appears clear without distortion.
Clarity and focus	A3	When focusing on objects at varying distances, the eyes can refocus quickly and see them clearly.
Comfort and stability	B1	After wearing the trial spectacles for a while, both eyes feel comfortable without obvious dizziness or ocular pressure.
Comfort and stability	B2	Both eyes feel coordinated, without double vision.
Comfort and stability	B3	When moving the head or the spectacles, the image in the visual field remains stable.
Environmental adaptation	C1	Vision remains clear without discomfort across indoor and outdoor environments.
Environmental adaptation	C2	Gait feels stable when walking on level ground.
Environmental adaptation	C3	Steps are clearly visible during stair negotiation without stumbling or missing a step.

Supplementary File S1 provides the complete English reporting translation, including instructions and response options, followed by the Mandarin source instrument. The English wording is author-generated for international reader comprehension and is not a cross-culturally validated English version of ASTWES.

### Limitations

This study was conducted in one center and restricted to adults aged 18–35 years trial-wearing conventional distance single-vision spectacles. This restriction reduced potential interference from age-related accommodative decline and near-add requirements, but the findings should not be extrapolated directly to older or presbyopic populations without further validation. Generalizability to other settings and lens designs also remains to be established. Patient-derived content validity was evaluated in a brief pre-pilot cognitive-interview sample and warrants confirmation in a larger, independently recruited patient group. Each subscale contains only three items, favoring brevity but reducing redundancy if an item performs differently in a new population (47, 48). Rasch or item-response modelling was not undertaken, so category thresholds, item information, and model-based local dependence were not evaluated. No repeat administration or comparator instrument was included; consequently, test-retest reliability, measurement error, responsiveness, and comparator-based construct validity were not estimated. The single-item 1-month satisfaction rating was a pragmatic anchor rather than a criterion standard, and independent threshold evaluation also remains necessary.

## Conclusion

ASTWES is a 9-item candidate PROM for immediate appraisal of distance single-vision spectacle trial wear. Pilot and formal analyses support three correlated subscales with acceptable internal consistency, while the overall mean score should remain secondary. The association with 1-month satisfaction is exploratory; independent patient-centered content evaluation, test-retest and comparator-based assessment, and external validation are required before routine use or threshold-based decisions.

## Data Availability

Unrestricted public sharing was not permitted by participant consent and ethics approval; requests for a de-identified dataset may be directed to the corresponding author and remain subject to institutional ethics and data-protection approval.
