# Supplementary material for: Development and preliminary psychometric evaluation of a spectacle trial-wear questionnaire for distance single-vision prescriptions
Source: Front Med (Lausanne). 2026 Oct 5;13:1935095. doi: 10.3389/fmed.2026.1935095 (PMC13677369; doi:10.3389/fmed.2026.1935095)
Supplement: Supplementary file 1 [file Supplementaryfile1.docx]

# Supplementary Material for "Development and Preliminary Psychometric Evaluation of a Spectacle Trial-Wear Questionnaire for Distance Single-Vision Prescriptions"

## Supplementary Table S1. Structured literature-search strategy used for item generation

| **Component** | **Specification** |
| --- | --- |
| Aim | To identify existing questionnaires, symptom descriptions, and clinically relevant task contexts for immediate adult spectacle trial wear. |
| Databases | PubMed, Web of Science, Embase, CNKI, Wanfang, VIP. |
| Time window | 1 January 2000 to 31 December 2024. |
| English search blocks | ("optometry" OR "spectacles" OR "dispensing" OR "trial wear" OR "trial frame") AND ("visual quality" OR "visual comfort" OR "symptom*" OR "adaptation" OR "satisfaction") AND ("questionnaire" OR "scale" OR "instrument" OR "patient-reported"). |
| Chinese search blocks | Chinese equivalents of the English spectacle, trial-wear, visual comfort, adaptation, satisfaction, questionnaire, and scale terms were used for CNKI, Wanfang, and VIP. |
| Included source types | PROMs or questionnaires relevant to visual symptoms or refractive care; spectacle non-tolerance studies; dispensing studies; clinically relevant case series describing symptoms or task difficulties during or shortly after spectacle wear. |
| Excluded source types | Studies focused only on surgery-related vision without relevance to spectacles; pediatric-only tools with no transferable item concepts; instruments aimed exclusively at long-term quality of life without immediate dispensing relevance; duplicate publications. |
| Use of evidence | Item generation, wording support, and construct framing only; this was not a standalone PRISMA-style systematic or scoping review. |

## Supplementary Table S2. Exploratory MLR measurement invariance across sex and age bands

Ordinal WLSMV subgroup models were attempted but could not be estimated because several item-by-group response categories were empty. The table below summarizes the sparse cells that led to an exploratory robust-MLR sensitivity analysis treating the five response categories as approximately continuous. These results should not be interpreted as confirmation of ordinal scalar invariance.

| **Grouping variable** | **Subgroup** | **n** | **Empty item-category cells** |
| --- | --- | --- | --- |
| Sex | Male | 198 | A1-1, A3-1, B1-1, B2-1, C1-1, C3-1 |
| Sex | Female | 195 | A1-1, A3-1, B1-1, C1-1, C3-1 |
| Age | 18-25 years | 166 | A1-1, A3-1, B1-1, C1-1, C2-1, C3-1 |
| Age | 26-35 years | 227 | A1-1, A3-1, B1-1, B2-1, B3-1, C1-1, C3-1 |

### S2A. Sex invariance (male vs female, formal sample n = 393)

| **Model** | **CFI** | **TLI** | **RMSEA** | **SRMR** | **Delta CFI** | **Delta RMSEA** | **Delta SRMR** |
| --- | --- | --- | --- | --- | --- | --- | --- |
| Configural | 0.976 | 0.964 | 0.055 | 0.037 | NA | NA | NA |
| Metric | 0.979 | 0.972 | 0.048 | 0.039 | +0.003 | -0.007 | +0.001 |
| Scalar | 0.976 | 0.972 | 0.049 | 0.041 | -0.003 | +0.001 | +0.002 |

### S2B. Observed age-band invariance (18-25 vs 26-35 years, formal sample n = 393)

| **Model** | **CFI** | **TLI** | **RMSEA** | **SRMR** | **Delta CFI** | **Delta RMSEA** | **Delta SRMR** |
| --- | --- | --- | --- | --- | --- | --- | --- |
| Configural | 0.986 | 0.979 | 0.042 | 0.033 | NA | NA | NA |
| Metric | 0.985 | 0.980 | 0.041 | 0.038 | -0.001 | -0.001 | +0.006 |
| Scalar | 0.988 | 0.986 | 0.035 | 0.039 | +0.003 | -0.006 | +0.001 |

## Supplementary Table S3. Diagnostics for the ordinal three-factor CFA

### S3A. Residual correlations with absolute value greater than 0.05

| **Item pair** | **Residual correlation** | **Absolute value** |
| --- | --- | --- |
| A1-C1 | 0.076 | 0.076 |
| A2-C2 | -0.072 | 0.072 |
| A1-B2 | -0.053 | 0.053 |
| A1-B3 | 0.052 | 0.052 |

### S3B. Largest modification indices

| **Proposed parameter** | **Modification index** | **Expected parameter change** |
| --- | --- | --- |
| Clarity factor loading on B2 | 3.370 | -0.246 |
| Residual covariance B1-B3 | 2.815 | -0.331 |
| Clarity factor loading on B3 | 2.798 | 0.280 |
| Residual covariance B1-B2 | 2.474 | 0.238 |
| Residual covariance A1-C1 | 2.236 | 0.312 |

*No modification index exceeded 10. The diagnostics were examined to contextualize the near-perfect incremental fit indices; no residual covariance or cross-loading was added post hoc.*

## Supplementary Note. English item wording

Table 8 in the main manuscript presents all nine items in English. Supplementary File S1 provides a complete English reporting form, including the context of use, instructions, response options, item wording, and scoring, followed by the Mandarin source instrument. The English wording is an author-generated reporting translation for reader comprehension only. Future English-language use should include forward-backward translation, expert bilingual reconciliation, and patient-based cognitive debriefing.

## Supplementary Table S4. Available patient debriefing audit trail

| **Stage** | **Patient sample and procedure** | **Main finding** | **Interpretation and decision** |
| --- | --- | --- | --- |
| Pre-pilot cognitive debriefing with open-ended concept prompts | 10 adults meeting the intended outpatient population and prescription-context criteria were recruited at the optometry clinic;; V2 questionnaire; combined think-aloud and verbal probing; <=15 min per interview; contemporaneous field notes and post-interview summaries | Participants reviewed item relevance and interpretation, response options, the immediate reference period, and task answerability. Discussions covered distance and near clarity, refocusing, binocular comfort, image stability, and ambulatory adaptation. | Concrete distance and near examples, the five response options, and the immediate trial-wear reference period were retained; feedback was incorporated into the research team's review of item wording, administration, and short-trial answerability. |
| Short-trial answerability review | The same 10 participants were asked whether the item content could be judged from the supervised trial. The route began outside the examination room, followed a relatively long corridor, included adjacent steps connecting the upper and lower floors, and extended into a hall with near and distant targets and areas of differing ambient brightness. | Participants encountered viewing-distance and brightness variation, level walking, and stair negotiation. An intern monitored task completion and confirmed completion on return. | The route and required task types were retained for pilot and formal administration. An exact walking distance, stair count, and repetition count were not prescribed. |

*A separate demographic and refractive-characteristics table was not retained for this 10-person formative group. These characteristics were not reconstructed retrospectively.*

## Supplementary Table S5. Item-level Delphi results and V1-to-V2 decision trail

### S5A. Item-level ratings

I-CVI was the proportion of experts assigning a relevance rating of 3 or 4. Importance CV was calculated as the sample standard deviation divided by the mean importance rating. Round 1 included 15 experts and round 2 included 14 experts.

| **V1 item** | **R1 I-CVI** | **R1 importance mean** | **R1 importance CV** | **R2 I-CVI** | **R2 importance mean** | **R2 importance CV** |
| --- | --- | --- | --- | --- | --- | --- |
| V1-01 | 0.933 | 3.27 | 0.356 | 0.929 | 4.50 | 0.169 |
| V1-02 | 1.000 | 4.20 | 0.258 | 0.929 | 4.57 | 0.141 |
| V1-03 | 0.933 | 3.93 | 0.263 | 1.000 | 4.21 | 0.231 |
| V1-04 | 0.800 | 3.20 | 0.395 | 0.929 | 2.93 | 0.390 |
| V1-05 | 1.000 | 4.13 | 0.221 | 1.000 | 4.57 | 0.141 |
| V1-06 | 0.933 | 3.80 | 0.267 | 1.000 | 3.00 | 0.392 |
| V1-07 | 1.000 | 4.07 | 0.286 | 0.929 | 4.43 | 0.192 |
| V1-08 | 1.000 | 4.20 | 0.273 | 1.000 | 4.43 | 0.171 |
| V1-09 | 1.000 | 4.33 | 0.208 | 0.929 | 4.07 | 0.225 |
| V1-10 | 1.000 | 4.27 | 0.242 | 1.000 | 4.50 | 0.169 |
| V1-11 | 0.933 | 3.80 | 0.318 | 0.786 | 3.29 | 0.221 |
| V1-12 | 0.933 | 3.27 | 0.271 | 0.786 | 2.93 | 0.313 |
| V1-13 | 1.000 | 4.33 | 0.167 | 1.000 | 4.21 | 0.190 |
| V1-14 | 0.867 | 3.33 | 0.387 | 1.000 | 3.00 | 0.346 |
| V1-15 | 0.933 | 3.80 | 0.285 | 1.000 | 4.14 | 0.186 |
| V1-16 | 1.000 | 4.13 | 0.272 | 1.000 | 4.43 | 0.171 |
| V1-17 | 0.867 | 4.27 | 0.207 | 1.000 | 4.50 | 0.115 |
| V1-18 | 0.800 | 4.00 | 0.283 | 1.000 | 4.36 | 0.171 |
| V1-19 | 1.000 | 4.20 | 0.205 | 1.000 | 4.50 | 0.169 |
| V1-20 | 1.000 | 3.87 | 0.291 | 1.000 | 4.43 | 0.192 |
| V1-21 | 0.933 | 3.53 | 0.259 | 0.857 | 3.29 | 0.221 |
| V1-22 | 0.867 | 2.87 | 0.435 | 1.000 | 3.29 | 0.303 |
| V1-23 | 1.000 | 3.00 | 0.356 | 1.000 | 3.00 | 0.346 |
| V1-24 | 0.867 | 3.13 | 0.338 | 0.714 | 3.43 | 0.248 |

### S5B. Item disposition

The English phrases below are concise reporting translations of the Mandarin V1 concepts, not a validated English questionnaire.

| **V1 item** | **Concise item concept** | **Decision and destination** | **Rationale** |
| --- | --- | --- | --- |
| V1-01 | Distant clarity without distortion | Retained as A1 | Core distance-clarity content |
| V1-02 | Intermediate-distance clarity | Retained as pilot A3; deleted after pilot testing | Distinct viewing-distance content was tested, but the item had weak item-total and factor performance and overlapped with retained distance, near, and refocusing content |
| V1-03 | Near clarity without diplopia | Retained as A2 | Core near-clarity content |
| V1-04 | Clarity during rapid distance switching | Deleted | Redundant with refocusing item and less directly answerable |
| V1-05 | Clarity during head movement | Merged into B3 | Represented by image-stability wording |
| V1-06 | Moving-target tracking | Deleted | Task dependent and redundant with dynamic-stability content |
| V1-07 | Binocular coordination without diplopia | Retained as B2 | Core binocular-comfort content |
| V1-08 | Absence of ocular fatigue or tension | Merged into B1 | Represented in the overall binocular-comfort wording |
| V1-09 | Absence of ocular pain or pressure | Merged into B1 | Combined into concise overall ocular-comfort wording |
| V1-10 | Absence of dizziness or vertigo | Merged into B1 and pilot B4 | Represented in the overall comfort item and the compound symptom item tested during the pilot |
| V1-11 | Absence of headache or pressure | Merged into pilot B4; deleted after pilot testing | Tested within the compound symptom item, which was removed for weak psychometric performance and heterogeneous content |
| V1-12 | Absence of neck discomfort | Merged into pilot B4; deleted after pilot testing | Tested within the compound symptom item, which was removed for weak psychometric performance and heterogeneous content |
| V1-13 | Rapid refocusing between distances | Retained as pilot A4 and renumbered final A3 | Core refocusing content; renumbered after deletion of pilot A3 |
| V1-14 | Stable clarity across distances | Deleted | Redundant with distance-specific clarity and refocusing items |
| V1-15 | Accurate spatial judgment on steps | Merged into C3 | Represented by stair-negotiation item |
| V1-16 | Stable vision with indoor/outdoor lighting change | Merged into C1 | Core environmental-adaptation content |
| V1-17 | Adaptation to bright/dim transitions | Retained as pilot C4; deleted after pilot testing | Tested as a candidate lighting-adaptation item but removed for weak item-total and factor performance and inconsistent short-trial answerability |
| V1-18 | Consistent vision across backgrounds | Merged into C1 | Redundant environmental-clarity content |
| V1-19 | Stable gait on level ground | Retained as C2 | Directly testable ambulatory content |
| V1-20 | Clear and stable stair negotiation | Merged into C3 | Directly testable stair content |
| V1-21 | Natural obstacle avoidance | Deleted | Task dependent and less feasible in a brief clinic trial |
| V1-22 | Stable vision during sit-to-stand | Deleted | Limited short-trial relevance and lower importance |
| V1-23 | No dizziness when bending or raising the head | Deleted | Redundant and less feasible to standardize |
| V1-24 | Continuous clear vision during turning | Deleted | I-CVI below criterion, limited short-trial answerability, and redundancy |

**Supplementary Table S6. De-identified characteristics of the Delphi panel**

Institution codes A-F are anonymized. Professional title was recorded as intermediate or senior under the corresponding institutional classification.

| **Expert ID** | **Field** | **Institution** | **Title** | **Experience, y** | **Ca** | **Cs** | **Cr** |
| --- | --- | --- | --- | --- | --- | --- | --- |
| E01 | Optometry | A | Senior | 15 | 0.84 | 0.87 | 0.85 |
| E02 | Optometry | B | Senior | 16 | 0.90 | 0.84 | 0.87 |
| E03 | Ophthalmology | A | Senior | 13 | 0.86 | 0.87 | 0.86 |
| E04 | Ophthalmology | D | Senior | 20 | 0.91 | 0.91 | 0.91 |
| E05 | Optometry | C | Senior | 18 | 0.94 | 0.81 | 0.88 |
| E06 | Optometry | C | Intermediate | 12 | 0.90 | 0.75 | 0.83 |
| E07 | Optometry | E | Senior | 14 | 0.88 | 0.96 | 0.92 |
| E08 | Dispensing | A | Intermediate | 19 | 0.90 | 0.91 | 0.90 |
| E09 | Dispensing | A | Intermediate | 15 | 0.86 | 0.86 | 0.86 |
| E10 | Dispensing | A | Senior | 22 | 0.89 | 0.84 | 0.87 |
| E11 | Optometry | F | Intermediate | 9 | 0.97 | 0.88 | 0.92 |
| E12 | Ophthalmology | D | Intermediate | 11 | 0.93 | 0.86 | 0.90 |
| E13 | Statistics/methodology | A | Senior | 14 | 0.91 | 0.92 | 0.92 |
| E14 | Ophthalmology | A | Senior | 19 | 0.93 | 0.91 | 0.92 |
| E15 | Statistics/methodology | B | Intermediate | 12 | 0.87 | 0.79 | 0.83 |
