# Supplementary material for: Development and preliminary psychometric evaluation of a spectacle trial-wear questionnaire for distance single-vision prescriptions
Source: Front Med (Lausanne). 2026 Oct 5;13:1935095. doi: 10.3389/fmed.2026.1935095 (PMC13677369; doi:10.3389/fmed.2026.1935095)
Supplement: Supplementary file 2 [file Supplementaryfile2.docx]

**Supplementary File S1. ASTWES English Reporting Translation and Mandarin Source Instrument**

**Part A. Complete English reporting translation**

**Adult Spectacle Trial-Wearing Experience Scale (ASTWES), Version 3**

**Context of use:** Adults aged 18-35 years complete the questionnaire after outpatient trial wear of the final candidate prescription for conventional distance single-vision spectacles.

**Instructions:** Based only on your experience during the current spectacle trial, indicate how well each statement describes your experience. Select one response for each item.

**Response options:** 1 = Strongly disagree; 2 = Disagree; 3 = Neither agree nor disagree; 4 = Agree; 5 = Strongly agree.

| **Domain** | **Item** | **English reporting translation** |
| --- | --- | --- |
| Clarity and focus | A1 | Viewing distant objects (e.g. signage at the end of a corridor) appears clear without distortion. |
| Clarity and focus | A2 | Viewing near objects (e.g. a smartphone screen) appears clear without distortion. |
| Clarity and focus | A3 | When focusing on objects at varying distances, the eyes can refocus quickly and see them clearly. |
| Comfort and stability | B1 | After wearing the trial spectacles for a while, both eyes feel comfortable without obvious dizziness or ocular pressure. |
| Comfort and stability | B2 | Both eyes feel coordinated, without double vision. |
| Comfort and stability | B3 | When moving the head or the spectacles, the image in the visual field remains stable. |
| Environmental adaptation | C1 | Vision remains clear without discomfort across indoor and outdoor environments. |
| Environmental adaptation | C2 | Gait feels stable when walking on level ground. |
| Environmental adaptation | C3 | Steps are clearly visible during stair negotiation without stumbling or missing a step. |

**Scoring:** Compute the mean of A1-A3, B1-B3, and C1-C3 as the three primary subscale scores. Do not calculate a subscale if any of its three responses is missing. An overall mean across all nine items may be reported only as a secondary descriptive summary and requires all nine responses. Higher scores indicate a more favorable immediate appraisal during trial wear. No fixed clinical cut-point has been validated.

**Translation status:** This English wording is an author-generated reporting translation supplied for international reader comprehension. It has not undergone a formal cross-cultural adaptation study and should not be treated as a validated English-language version.

**Part B. Mandarin source instrument**

**成人框架眼镜试戴体验评估量表（ASTWES）V3**

**本研究适用情境：**18-35岁成人在门诊试戴最终拟配的远用单光框架眼镜处方后填写。

**填写说明：**请根据本次试戴过程中的实际感受，判断下列描述与您的情况是否相符。每个条目只能选择一个答案。

**评分：**1 = 非常不同意；2 = 不同意；3 = 一般；4 = 同意；5 = 非常同意。

| **维度** | **条目** | **中文原始条目** |
| --- | --- | --- |
| 清晰度与对焦 | A1 | 看远处（如走廊尽头的指示牌）感觉清晰，没有变形。 |
| 清晰度与对焦 | A2 | 看近处（如手机屏幕）感觉清晰，没有变形。 |
| 清晰度与对焦 | A3 | 在注视不同距离的物体时，眼睛能快速对焦，看清物体。 |
| 舒适性与稳定 | B1 | 长时间佩戴后左右眼均感到舒适，无明显头晕或眼胀。 |
| 舒适性与稳定 | B2 | 两眼感觉协调，不会出现重影。 |
| 舒适性与稳定 | B3 | 移动头部或眼镜时，视野中的图像保持稳定。 |
| 环境适应 | C1 | 在室内外不同环境中，视物清晰，不会产生不适。 |
| 环境适应 | C2 | 在平地上行走时，感觉步伐稳定。 |
| 环境适应 | C3 | 上下楼梯时，能看清台阶，不会绊脚或踩空。 |

**计分说明：**A1-A3、B1-B3和C1-C3分别取平均值，作为3个主要维度分。任一维度缺失1个条目时不计算该维度分。9个条目均完成时，可计算总题均分作为次要描述性指标。分数越高表示本次试戴的即时体验越好。目前尚无经过验证的固定临床界值。
